# Contact tracing apps: an ethical roadmap

**DOI:** 10.1007/s10676-020-09548-w

**Published:** 2020-09-29

**Authors:** Marjolein Lanzing

**Affiliations:** Interdisciplinary Hub for Security, Privacy and Data Governance, Faculty of Philosophy, Theology and Religious Studies, Radboud University Nijmegen, IHub 19th floor, room 19.06, Houtlaan 4, 6525 Nijmegen, XZ The Netherlands

**Keywords:** Contact tracing apps, Privacy, Data monopolists, Coercion, Solidarity

## Abstract

This research statement presents a roadmap for the ethical evaluation of contact tracing apps. Assuming the possible development of an effective and secure contact tracing app, this roadmap explores three ethical concerns—privacy, data monopolists and coercion- based on three scenarios. The first scenario envisions and critically evaluates an app that is built on the conceptualization of privacy as anonymity and a mere individual right rather than a social value. The second scenario sketches and critically discusses an app that adequately addresses privacy concerns but is facilitated by data monopolists such as Google and Apple. The final scenario discusses the coerced installation and use of a privacy-friendly, independently developed contact tracing app. The main worry is coercion through societal exclusion and limited societal participation. The statement concludes with three suggestions for designing an ethical contact tracing app and a research agenda.

## Roadmapping beyond privacy: two approaches for mapping ethical considerations involving contact tracing apps

As many countries across the globe are struggling with the Covid-19 virus, a discussion is taking place about the possible use of (a wide variety of) contact tracing apps. The goal is to gain insight in the spread of the corona-virus which, in many cases, requires location data and biometric information. Most concerns about these apps in the discussion focus on privacy as an individual right to control over one’s information (Davidson 2020; Hao 2020; Timberg and Harwell 2020; Wetsman 2020). However, we believe that this discussion should be broadened to include other ethical considerations and a richer understanding of privacy as a public value.

Lanzing and Siffels present two research statements that contribute to the discussion by offering considerations ‘beyond privacy’ when evaluating the development and implementation of contact tracing apps. The first,

‘*Contact tracing apps: an ethical roadmap*’, presents a roadmap for the ethical evaluation of contact-tracing apps. It raises three ethical concerns—privacy, Big Tech dependency and coercion-by exploring three scenarios (Lanzing 2020, this issue). The second, ‘*Beyond Privacy vs. Health: a justification analysis of contact-tracing apps debate in the Netherlands*’, shows how a justification analysis of the debate about contact tracing apps, using the framework developed by Luc Boltanski and Laurent Thevenot, can enable us to recognize a plurality of common goods at stake (Siffels 2020, this issue).

Both statements are part of the ‘Digital Good’ project, an interdisciplinary research project that focuses on the disruption of health as we move into the digital era. The project investigates ways of approaching the digitalization of health from a standpoint of the common good, rather than one of individual privacy. Its aim is to look for governance frameworks that foreground collective welfare and public values, while acknowledging a plurality of conceptions of the common good at work in the digitalization of health.

## Contact tracing apps: an ethical roadmap

Many countries across the globe are currently developing (or already using) contact tracing apps (Meaker and Tokmetzis 2020). The contact tracing apps are smartphone applications that track whether someone had contact with a person infected with Covid-19. While there are many varieties, most apps require sensitive personal information such as one’s geo-location and biometric information. Legal scholars, ethicists and activists have voiced their concerns regarding the responsible use of data in terms of security, fair data sharing practices, voluntariness and privacy in various reports and manifestos (Ienca and Vayena 2020; Nuffield Council on Bioethics 2020; Soetenhorst 2020; https://www.veiligtegencorona.nl/). Public officials and developers have responded to (some of) these concerns by promising voluntary use and anonymity to ensure privacy (Miserus and Verhagen 2020).

This research statement provides an ethical roadmap for the development and implementation of contact tracing apps beyond privacy-as-anonymity.^1^ It contributes by answering the following research question: ‘Assuming the possibility of an effective and secure contact tracing app: what are the possible ethical objections?’ The roadmap explores three ethical concerns—privacy, data monopolists and coercion- based on three scenarios.^2^ The first scenario envisions and critically evaluates an app that is built on the conceptualization of privacy as anonymity and a mere individual right rather than a social value. The second scenario sketches and critically discusses an app that adequately addresses privacy concerns but is facilitated by data monopolists such as Google and Apple. The final scenario discusses the coerced installation and use of a privacy-friendly, independently developed contact tracing app. The main worry is coercion through societal exclusion and limited societal participation. The paper concludes with three suggestions for designing an ethical contact tracing app.

## Privacy as a social condition

The first scenario is one in which privacy is narrowly conceptualized as anonymity. Most public officials and developers promise anonymous data in order to address privacy concerns. For instance, the Dutch Privacy Protection Office (Autoriteit Persoonsgegevens) expressed that ‘anonymity is key’ when implementing a contact tracing app (Miserus and Verhagen 2020). At the same time, politicians and public officials emphasize the importance of public health over privacy—implying that privacy is an individual right that should be sacrificed (Hao 2020; McGee et al. 2020). There are problems with both the conceptualization of privacy as anonymity and the dichotomy between privacy and public health that portrays privacy as an (merely) individual right.

First, privacy is not synonymous with anonymity. Privacy entails that one can *choose* what they want to share (and with whom) (Nissenbaum 2010; Westin 1969). We may *want* to share certain information with certain parties. The question is whether the app involves (future) parties that users can trust with their information. Anonymity entails that parties that you do not want to access your data, *can* access your data but simply will not know who the data belongs to.

Moreover, ‘health versus privacy’ is a false contradiction. Health and privacy are not necessarily mutually exclusive. Sharing one’s biometric information with a health professional or a research institute in order to run a COVID-19 test is not a violation of privacy. It becomes a violation when this information is shared with parties that should not have access to this data.

Third, the contradiction presupposes that citizens should sacrifice an individual right, for a collective good. However, privacy is social (Roessler and Mokrosinska 2015). It is not an individual luxury but an important condition for a free society: a society in which one cannot be arbitrarily manipulated by the government, one’s employer, a health insurer or Big Tech (Susser et al 2018). Without privacy, citizens are all to a certain extent vulnerable to unwanted interference. It is therefore an act of solidarity to stand up for the right to privacy. Citizens and representatives of liberal democratic constitutional states must therefore carefully monitor the developments of contact tracing apps.^3^

Finally, privacy-as-anonymity is insufficient to safeguard these social dimensions. Anonymity is a relative concept. From ‘anonymous’ data, one can deduce information about groups and individuals. Information is not merely personal. By contributing data, even anonymously, one also reveals information about other people (Barocas and Levy 2020). Moreover, privacy does not only have an informational, but also a decisional dimension (Lanzing 2018). One can interfere with individuals’ behavior and choices based on ‘group’ data. In the case of contact tracing apps, Marijn Sax suggested that one may receive a notification that tells one to ‘stay inside’ or ‘get tested’ based on anonymized geo-location and biometric data of the people in one’s neighborhood.^4^

## The googlization of health crisis management

The second scenario assumes that the contact tracing app is effective and privacy-sensitive.^5^ Can there still be something wrong? Tamar Sharon argues that privacy is not the only concern (Sharon 2016, 2020). There might still be something wrong when these apps are developed by data monopolists such as Google and Apple (Sharon 2020, this issue). Google and Apple have developed technology for a contact tracing app suitable for iPhones and smartphones. Using a Bluetooth signal, a log is created that indicates who the user has been in contact with. Infections are monitored on a central server of a health authority. Governments can use the Google/Apple tools to develop their own app and run it on the software of iPhones and smartphones. Apple and Google promise security and privacy. For example, they only support one contact tracing app per country. Also, this app can only be used for controlling the virus and not for advertisements. Only health authorities can access the technology. Finally, it is a decentralized system that stores one’s personal data on one’s phone.

Sharon raises several concerns regarding the ‘Googlization of Health Crisis Management’ (Sharon 2016, 2020). She warns that this is yet another aspect of our daily lives (in addition to social domains such as education, transport and smart cities) in which society becomes dependent on monopolists. Companies like Google have been investing in the health sector and collecting health data for years. By encouraging users to use a technology made by Google to contain the virus, society welcomes a monopolist in a crucial part of public health crisis management (Klein 2020; Morozov 2020). This allows these corporations to shape these domains. Not on the basis of democratic values, but on the basis of their own, possibly commercial, interests. Once citizens become dependent on these companies, they lose their grip on what they want these social domains to look like (Sharon (2020)).

## Coercion

The final scenario is one in which a contact tracing app is not developed by data monopolists but an independent non-profit party. The ethical concern that remains in this scenario is coercion. Apart from feasibility—not everyone owns or is able to use a smartphone—the coerced use of an app is at odds with a liberal democratic constitutional state. In a democracy, the autonomy of citizens is respected by allowing citizens to make their own decisions as much as possible. Voluntary use of the app is therefore a key condition.

But there are more forms of coercion that governments should protect citizens from, which I will refer to as societal coercion. What if employers, restaurants or schools only grant access when someone can prove that they are not infected with Covid-19 with a contact tracing app? There will also be people who choose not to download the app. This should be possible without being excluded from work, school or public transport. It is unfair if social participation and inclusion depend on the installation of a contact tracing app (Floridi 2020). Therefore, it is necessary that the government develops policies against societal coercion.

While this argument is powerful on its own, it becomes all the more convincing when the contact tracing app involves surveillance by the government or a corporation that forces people (implicitly) to share their data. Moreover, it becomes particularly exploitative when the data that is shared by users for the sake of ‘saving lives’ is used for ‘privatized interventions from which communities from whom the data was generated are shut out’ (Nuffield Council on Bioethics 2020, p. 187).

## Three suggestions for policy and future research

In sum, there are three ethical concerns that are important to take into account when designing a contact tracing app. *First*, when developing an app, developers and policy makers should understand that anonymity should not be equated with privacy. It is inadequate for capturing the social value of privacy and protecting people against unwanted interference.

*Second*, governments should let independent, non-commercial parties develop the app and underlying infrastructure. When we increasingly rely on data monopolists in the health (crisis management) domain, citizens are increasingly less able to shape this domain via democratic procedures and based on public values.

*Third*, the app should not be coerced in any way. Neither by direct coercion, nor by making it a precondition for social participation. The government should develop policies around the app in order to prevent this form of coercion.

It is important that these three policy suggestions based on ethical concerns are included in the design and implementation procedure of contact tracing apps. Moreover, a future research agenda in the ethics of technology should include in-depth investigations of privacy as a public value; the increasing dependency on Big Tech in society and decentralized forms of coercion by means of technology.

In times of crisis, we are more inclined and willing to curtail our civil liberties. Citizens must be alert to the fact that these measures and resources are exceptions in an emergency. They should not become the standard when the crisis is over. Experience shows that after a crisis society often lingers in policies and behaviors that were initially designed for emergency purposes (Ross 2020).

The development and implementation of a contact tracing app should not be conceived as a societal experiment (Lucivero et al 2020; Van de Poel 2013). Technology can sometimes seem to be an easy and quick solution to social problems, while it can have social consequences that are difficult to oversee or reverse. A contact tracing app that has not been designed and implemented based on public values, a democratic procedure and under strict conditions may undermine trust and solidarity in the long run.

## Footnotes

1 This paper is based on: Lanzing (2020).

2 Of course, this is not an exhaustive list. Concerns such as exploitation, trust, transparency and democratic accountability are some of the concerns that flow from the scenarios.

3 See also the interview with former Member of the European Parliament Marietje Schaake (Modderkolk 2020).

4 See Marijn Sax’ response to contact tracing apps and privacy debate in the Netherlands in: Breebaart (2020).

5 For this scenario we assume that an app is developed based on a social conceptualization of privacy or we assume that there are good reasons to accept ‘privacy-as-anonymity’.
